# *Pseudomonas rhodesiae* HAI-0804 suppresses *Pythium* damping off and root rot in cucumber by its efficient root colonization promoted by amendment with glutamate

**DOI:** 10.3389/fmicb.2024.1485167

**Published:** 2024-11-05

**Authors:** Kasumi Takeuchi, Masayo Ogiso, Arisa Ota, Kentaro Nishimura, Chihiro Nishino, Yasuhiro Omori, Mitsunori Maeda, Ryousuke Mizui, Homare Yamanaka, Tomokazu Ogino, Shigemi Seo

**Affiliations:** ^1^Institute of Agrobiological Sciences, National Agriculture and Food Research Organization, Tsukuba, Japan; ^2^Field Research Department, Odawara Research Center, Nippon Soda Co., Ltd., Shizuoka, Japan; ^3^Development Department, Agro Products Division, Nippon Soda Co., Ltd., Tokyo, Japan; ^4^Nisso Field Service Co., Ltd., Shizuoka, Japan

**Keywords:** pseudomonads, rhizosphere, disease control, biofilm, root colonization, glutamate

## Abstract

Plant diseases caused by soil-borne fungi and oomycetes significantly reduce yield and quality of many crops in the agricultural systems and are difficult to control. We herein examine *Pseudomonas rhodesiae* HAI-0804, a bacterial biological control agent that was originally developed for control of bacterial diseases on the surface of vegetables, and assessed its efficacy at controlling soil-borne diseases caused by oomycetes. Strain HAI-0804 did not exhibit detectable antibiotic activity toward *Pythium ultimum*, a causal agent of damping-off and root rot; however, it effectively protected against *Pythium* damping-off and root rot in cucumber. Exogenous glutamate enhanced the efficacy of biocontrol, the production of siderophore pyoverdine, root colonization in cucumber plants, and the ratio of biofilm formation to planktonic cells. The epiphytic fitness of strain HAI-0804 appears to contribute to plant protection efficacy against a broad spectrum of pathogens for both above-ground plant parts and the rhizosphere.

## 1 Introduction

Soil-borne diseases, such as damping-off and root rot caused by species of *Pythium*, *Fusarium*, and *Rhizoctonia*, significantly reduce the yields of the agricultural systems of many crops and are difficult to control because these pathogens survive for long periods in soil (Janvier et al., 2007; Arora et al., 2021; Wille et al., 2019). The excessive use of synthetic fungicides will result in the emergence of resistant pathogens. This has prompted intensive research on the development of biocontrol agents that utilize beneficial microorganisms represented by plant growth-promoting rhizobacteria (PGPR) (Weller, 1988). Many PGPR isolates in the genera *Bacillus* and *Pseudomonas* have been employed as biocontrol agents (Niu et al., 2020; Dimkić et al., 2022). These strains have been shown to directly suppress the growth of plant pathogens via the production of antibiotic secondary metabolites in the rhizosphere. For example, root-colonizing fluorescent pseudomonads classified into the *Pseudomonas fluorescens* group produce a number of antibiotic secondary metabolites and extracellular enzymes that contribute to the suppression of pathogenic fungi, nematodes, and insects; therefore, they are regarded as effective biocontrol strains against plant diseases (Haas and Keel, 2003). Recent genomic studies on root-colonizing fluorescent pseudomonads revealed that the distribution of several biosynthetic gene clusters is specific to strains (Takeuchi et al., 2023a). Therefore, antibiotic secondary metabolites might not be a major contributor to the biocontrol efficacy in some cases.

Other than the direct suppression of the growth of plant pathogens, PGPR play versatile roles in nutrient cycling and increase the productivity of crops (Santoyo et al., 2021). PGPR enhance plant productivity by increasing the uptake of nutrients by the rhizosphere. PGPR also confer protection against pathogens through indirect mechanisms such as the induction of systemic resistance, which is regulated by phytohormones, such as salicylic acid (SA) and jasmonic acid (JA) (Iavicoli et al., 2003; Ahn et al., 2007). The development of biofilms on root surfaces following root colonization by PGPR is one of the main mechanisms that promote plant health by exerting properties such as physical adhesive strength on root surfaces, thereby creating a physical barrier against pathogens and abiotic stresses (Flemming and Wingender, 2010). Furthermore, the dense bacterial community that resides in biofilms confers the advantage of expressing biocontrol factors in a quorum sensing-controlled manner (Mukherjee and Bassler, 2019).

In terms of effective and ecological applications of biocontrol agents, evaluations of plant commensal pseudomonads have led to advances in plant protection research. The modes of action of biocontrol agents need to be clarified in order to increase the plant protection efficacy of strains. Several pseudomonad strains have been increasingly marketed as biocontrol agents. Among them, the genome of *P. fluorescens* strain A506, which is sold as BlightBan^®^ A506 (Nufarm Americans, Burr Ridge, IL, United States) for the suppression of the bacterial disease fire blight in pear and apple orchards in the United States and Canada, was elucidated in a comparative genomic project on biocontrol pseudomonads (Loper et al., 2012), providing some distinct features of this strain. Furthermore, *P. chlororaphis* MA 342, which is used for seed coating to suppress seed-borne pathogens, and the products marketed as Cedomon^®^ and Cerall^®^ (BioAgri AB, Sweden) in Europe are producers of rhizoxin analogs (Johansson and Wright, 2003). Other examples of commercial products based on pseudomonads are Spot-Less^®^ (*P. aureofaciens* Tx-1; Turf Science Laboratories, Carlsbad, CA, United States), AtEze^®^ (*P. chlororaphis* 63–28; Turf Science Laboratories, Carlsbad, CA, United States), and Bio-save^®^ (*P. syringae* ESC-10, and ESC-11; Jet Harvest Solutions, Longwood, FL, United States). These formulated *Pseudomonas* spp. display additional activities because they may also be used as bioinsecticides or as rhizobacteria with plant growth-promoting activity (Kupferschmied et al., 2013).

The strain *P. rhodesiae* HAI-0804 was originally isolated from the surface of lettuce leaves and was developed as the active component of the biological control agent marketed as Masterpiece^®^ WP (Nippon Soda Ltd., Japan) against bacterial diseases on the surface of above-ground plant parts, such as vegetables, in Japan (Maeda, 2012, 2013; Maeda et al., 2014). In the present study, we examined the epiphytic fitness of strain HAI-0804 on the roots of cucumber and demonstrated its efficacy as a biocontrol agent against *Pythium* damping-off and root rot in cucumber through its effective colonization of root surfaces. Strain HAI-0804 did not exhibit any detectable antibiotic activity against other microbes. Therefore, the mechanisms by which diseases are suppressed involve factors other than antibiotic activity. We investigated root colonization on cucumber root surfaces the biofilm-forming abilities of this strain. We also investigated the effect of exogenous glutamate, which exerted positive effects on the plant protection efficacy of *P. protegens* strains in our previous study (Takeuchi et al., 2023b). We observed that the properties of root colonization and biofilm formation by the strain HAI-0804 were enhanced by exogenous glutamate, which correlated with the plant protection efficacy of this strain.

## 2 Materials and methods

### 2.1 Bacterial strains and growth conditions

The bacterial strains and plasmids used in the present study are listed in Table 1. *Pseudomonas* spp. were routinely grown in nutrient yeast broth (NYB; 2.5% [w/v] nutrient broth, 0.5% [w/v] yeast extract) with shaking or on nutrient agar plates (4% [w/v] blood agar base, 0.5% [w/v] yeast extract). In other assays, bacteria were grown in modified glycerol-casamino acid medium (GCM; Maurhofer et al., 1998) without the amendment of minerals. Tetracycline was amended at 100 μg/mL when required. The inoculation temperature for *Pseudomonas* sp. was 30°C.

**Table 1:** Bacterial strains and plasmids used in the present study.

Strain or plasmid	Description	Source or reference
Strains		
*Bacillus subtilis*		
M168	Wild type	DSM, NBRC
*Pseudomonas chlororaphis*		
MAFF302536	Wild type	NARO Genebank
*Pseudomonas fluorescens*		
MAFF301597	Wild type	NARO Genebank
NBRC14160^T^	Wild type	RIKEN BRC
*Pseudomonas protegens*		
Cab57	Wild type	Takeuchi et al. (2014)
*Pseudomonas rhodesiae*		
HAI-0804	Wild type	NITE (FERM P-21025)
JCM11940^T^	Wild type	RIKEN BRC
Plasmids		
pME6031	pACYC177-pVS1 shuttle vector; Tc^r^	Heeb et al. (2000)
pME7402	Transcriptional *rsmZ-gfp* fusion; Tc^r^	Dubuis et al. (2006)

### 2.2 Plant disease suppression assays

The biocontrol activity of *P. rhodesiae* HAI-0804 was evaluated as follows. Cucumber (*Cucumis sativus* L. cv. Tokiwajibai) seeds were pre-conditioned on filter paper sufficiently wetted with distilled water at 26°C for 24 h and then sown in vermiculite in 125-cm^3^ plastic pots (5 × 5 × 5 cm). Three cucumber seedlings were subsequently planted in individual pots containing 50 mL of vermiculite. *Pythium ultimum* (reidentified as *Globisporangium oryzicola*; Uzuhashi et al., 2017) MAFF425494 was added as a millet-seed inoculum at 5 g per kg of vermiculite before planting. Strain HAI-0804 was grown in NYB and added to vermiculite as a suspension (5 mL per pot) of cells washed once in sterile distilled water to set an OD_600_ of 0.01 or 0.1. To evaluate the effect of exogenous glutamate, 8 mL of 1 or 10 mM glutamate solution was then added to vermiculite as the glutamate treatment. Control pots received the same amount of sterile water. Seedlings were covered with 15 mL of untreated vermiculite. Microcosms and cucumber seedlings were placed in a growth chamber set to long-day growth conditions (16 h light/8 h dark) and then incubated at 26°C with 60% relative humidity. Watering was performed every 3–4 days and glutamate was added instead of water as the glutamate treatment after an incubation for 1 week. After an incubation for 2 weeks, the number of surviving plants was counted and shoot and root fresh weights in each pot were measured, based upon which the biocontrol activity of each strain was evaluated. The means of the same experiment performed in duplicate are shown in Table 2. The results obtained at a lower inoculum intensity of pseudomonad are shown in Table 3. To evaluate the effect of HAI-0804 itself on the growth of cucumber, the results obtained without the addition of *Pythium* are shown in Supplementary Table S2. An analysis of variance (ANOVA) was performed on the results of both experiments to identify a trial-by-treatment interaction, which showed that data from two independent experiments may be pooled. Tukey’s HSD test was conducted to examine the significance of differences in means (at *p* ≤ 0.05). R software (version 4.1.1) was employed for statistical analyses.

**Table 2:** The suppression of *Pythium* damping-off and root rot in cucumber by *Pseudomonas rhodesiae* HAI-0804.

Bacterial strain and glutamate added	*Pythium* added*	Surviving plants per pot (%)**	Shoot fresh weight per pot (g)**	Root fresh weight per pot (g)**
None	−	100^a^	1.16^a^	0.38^a^
None	+	22^d^	0.18^c^	0.07^b^
HAI-0804	+	47^cd^	0.38^b^	0.12^b^
HAI-0804 + Glu1	+	75^ab^	0.53^b^	0.20^c^
HAI-0804 + Glu10	+	61^bc^	0.50^b^	0.20^c^

* *Pseudomonas rhodesiae* HAI-0804 was added at 10^7^ CFU per gram of vermiculite (25 g of vermiculite per pot) contained within 125-ml pots, after planting three cucumber seedlings per pot. *P. ultimum* was added as a millet-seed inoculum at 5 g/kg of vermiculite before planting. Plants were harvested after 14 days. ** Data represent the means of two individual repetitions of the same experimental set-up, with 6 replicates (pots containing three cucumber plants) per treatment in each experiment and 12 replicates (pots) in total. Means within the same column followed by different letters (a-d) are significantly different (*P* < 0.05) according to Tukey’s HSD test.

**Table 3:** The suppression of *Pythium* damping-off and root rot in cucumber by *Pseudomonas rhodesiae* HAI-0804 at a lower inoculum intensity and the effects of the addition of glutamate.

Bacterial strain and glutamate added	*Pythium* added*	Surviving plants per pot (%)**	Shoot fresh weight per pot (g)**	Root fresh weight per pot (g)**
None	−	100^a^	1.20^a^	0.40^a^
None	+	44^c^	0.38^c^	0.13^c^
HAI-0804	+	72^b^	0.65^b^	0.21^bc^
HAI-0804 + Glu1	+	89^ab^	0.85^b^	0.26^b^

* *Pseudomonas rhodesiae* HAI-0804 was added at 10^6^ CFU per gram of vermiculite (25 g of vermiculite per pot) contained within 125-ml pots, after planting three cucumber seedlings per pot. *P. ultimum* was added as a millet-seed inoculum at 5 g/kg of vermiculite before planting. Plants were harvested after 14 days. ** Data represent the means of two individual repetitions of the same experimental set-up, with 6 replicates (pots containing three cucumber plants) per treatment in each experiment and 12 replicates (pots) in total. Means within the same column followed by different letters (a-d) are significantly different (*P* < 0.05) according to Tukey’s HSD test.

### 2.3 Plant root colonization assay

Three sterile-grown cucumber seedlings (48 h old) were planted in 150 mL of non-sterile, commercial soil (Supermix, Sakata Co., Ltd., Japan) (pH5.0–6.0, N:150–260 P:70–160 K:190–320 mg/L) in 200-ml plastic pots. Strain HAI-0804 was added to soil as a suspension (10 mL per pot, 1.5 × 10^9^ CFU) of cells in the early stationary phase and washed once in sterile distilled water to set an OD_600_ of 0.15. Then 10 mL of 10 mM glutamate was added to the soil as the glutamate treatment. Control pots received the same volume of sterile water. Seedlings were covered with 15 mL of untreated soil. They were placed in a growth chamber set to long-day growth conditions (16 h light/8 h dark) and then incubated at 26°C. Watering was performed every 3–4 days and 10 mM glutamate was added instead of water as the glutamate treatment every week. Following an incubation for 30 days, sterile ultrapure water was used to thoroughly rinse the whole roots. Roots were then suspended in 15 mL of saline (0.9% NaCl) in 50-ml conical tubes. Bacteria were extracted by vigorous vortexing and then counted using plating dilutions.

### 2.4 Evaluation of antimicrobial activity

In the present study, *Bacillus subtilis* M168 and *P. ultimum* MAFF425494 were employed as reporter strains to monitor the antimicrobial activities of *P. rhodesiae* HAI-0804 and *P. protegens* Cab57 (Takeuchi et al., 2014). Antimicrobial activities of *P. chlororaphis* MAFF302536 and *P. fluorescens* MAFF301597 toward *P. ultimum* were also tested. *B. subtilis* was employed to monitor the production of antibiotics produced by *Pseudomonas* spp. by evaluating the halo size (Takeuchi et al., 2009; Takeuchi et al., 2014). Cultures of *Pseudomonas* spp. in the early stage of the stationary phase were adjusted to OD_600nm_ = 1.0 (1 × 10^9^ CFU/mL) for the assay with *B. subtilis*, and 5-μl samples were spotted onto a plate with modified GCM without the amendment of minerals. After an incubation at 28°C overnight, cells were killed by 254-nm UV irradiation for 5 min on a transilluminator. An overlay of *B. subtilis* revealed antibiotic production by growth inhibition zones. Cultures of *Pseudomonas* spp. were adjusted to OD_600nm_ = 1.5 for the assay with *P. ultimum*, and 20-μl samples were streaked around the edge of a potato dextrose agar plate, and an inoculum of *P. ultimum* was transferred to the center of the plate. The plate was incubated at room temperature until *P. ultimum* reached the edge of the plate.

### 2.5 Biofilm formation assay

Bacterial cells grown overnight in NYB were washed once in saline and OD_600_ in saline was adjusted to 3.0. One-microliter aliquots were added to each well of a 96-well flat-bottomed polystyrene plate (IWAKI Co., Ltd., Japan) containing modified GCM without the amendment of minerals (200 μL per well) and supplemented with or without amino acids (glutamate, histidine, glutamine, glycine, leucine, phenylalanine, lysine, valine, isoleucine, tryptophane, alanine, and methionine). Cultures were incubated without agitation at room temperature for 24 h in a covered container that contained a water-soaked paper towel, which prevented evaporation. OD_600_ values in 100 μL of planktonic cell cultures were measured. Biofilms were stained by adding 50 μL of a 0.5% solution of crystal violet to each well of the plate, incubating the plate at room temperature for 15 min, and then rinsing the plate thoroughly with water. To quantify biofilm growth, 400 μL of 95% ethanol was added to each well to desorb the stained biofilm followed by 600 μL of water, and the amount of crystal violet that bound to each biofilm was measured directly as absorbance at 600 nm (A_600_) (Takeuchi, 2018).

### 2.6 Measurement of pyoverdine concentrations

The concentration of siderophore pyoverdine produced by *P. rhodesiae* HAI-0804 was estimated spectrophotometrically as the absorbance at 405 nm (Taguchi et al., 2010). Bacterial cells grown overnight in NYB were washed once in saline and 150-μl aliquots were added to 50-ml flasks containing 15 mL of modified GCM without the amendment of minerals, supplemented with or without glutamate. After an incubation at 180 rpm at 28°C overnight, the absorbance of the supernatant was measured at 405 nm (A_405_) for the quantification of pyoverdine production. OD_600_ values in each cell cultures were also measured.

### 2.7 Microscopy

Three sterile-grown cucumber seedlings (48 h old) were planted in 100-ml flasks containing 35 mL of vermiculite. Strain HAI-0804 harboring the GFP-expressing plasmid pME7402 was added to vermiculite as a suspension (4 mL per pot) of cells washed once in sterile distilled water to set an OD_600_ of 0.1. Seedlings were covered with 5 mL of untreated vermiculite, placed in a growth chamber set to long-day growth conditions (16 h light/8 h dark), and then incubated at 26°C. After an incubation for 2 weeks, sterile ultrapure water was used to thoroughly rinse whole roots. Cucumber roots were then aseptically cut into 1-cm-long pieces from the tip to the upper part of the root for microscopic observations.

To detect single bacteria of strain HAI-0804 on the roots, laser scanning confocal microscopy was performed using a TCS SP5 instrument (Leica). Fluorescence was excited with an argon laser at 488 nm and detected at wavelengths of 500–520 nm. Images were obtained using a fluorescence microscope (DM6000B; Leica) equipped with a confocal laser scanning unit (CSU-X1; Yokogawa Electric, Tokyo, Japan), laser units (Sapphire 488 and 561 nm; Coherent, Santa Clara, CA), a dichroic mirror (DM-405/488/561), and emission filters (GFP, EM-520/35). Images were processed and arranged using MetaMorph software (Molecular Devices LLC, Sunnyvale, CA).

### 2.8 Total RNA extraction and quantitative real-time polymerase chain reaction

Roots harvested from three cucumber plants were combined as one biological replicate and subjected to the extraction of total RNA using TRIzol reagent (Invitrogen) in accordance with the manufacturer’s instructions. Quantitative real-time PCR using total RNA was performed in a two-step reaction using a SYBR Green kit (Bio-Rad, Hercules, CA, United States) and specific primers (Supplementary Table S1) as previously described (Seo et al., 2016). The expression levels of the clathrin adaptor complex subunit were used to normalize those of the target genes (Tingting et al., 2022). Dunnett’s test was used for Supplementary Figure S1. Statistical analyses were conducted using R version 4.1.2.

### 2.9 Measurement of phytohormones

Roots harvested from three cucumber plants were combined as one biological replicate and used to measure phytohormones SA and JA. The extraction and quantification of SA and JA were performed as previously described (Seo et al., 2016; Kishi-Kaboshi et al., 2018; Murata et al., 2019). Dunnett’s test was used for Supplementary Figure S2. Statistical analyses were conducted using R version 4.1.2.

## 3 Results

### 3.1 Strain HAI-0804 exhibited plant protection efficacy against *Pythium* damping-off and root rot in cucumber

To investigate the biocontrol activity of strain HAI-0804 in the rhizosphere in a natural habitat, we adopted a cucumber-*P. ultimum* pathosystem, which enabled us to evaluate plant protection efficacy by measuring root and shoot weights. Strain HAI-0804 exhibited plant protection efficacy, increasing shoot fresh weights over that of the *Pythium* control (without addition of strain HAI-0804), with 95% confidence, whereas the number of surviving plants was not significantly different at this confidence level (Table 2).

### 3.2 Exogenous glutamate enhanced the efficacy of biocontrol in cucumber plants

In our previous study, exogenous glutamate exerted positive effects on the plant protection efficacy of *P. protegens* strains Cab57 and CHA0, whereas glutamate without addition of these strains had no effect on the suppression of disease (Takeuchi et al., 2023b). We examined the effects of adding glutamate to strain HAI-0804. Based on our previous findings and in consideration of initial water absorbed in vermiculite, we applied 10 mM of glutamate to vermiculite to reach a final concentration of approximately 5 mM in the rhizosphere (Takeuchi et al., 2023b). We also applied glutamate at a lower concentration by one order (1 mM). The addition of glutamate at both concentrations enhanced the plant protection efficacy of strain HAI-0804. Glutamate with the strain HAI-0804 also markedly affected root fresh weights (Table 2). The treatment with strain HAI-0804 and glutamate did not affect the fresh weight of cucumber grown without the addition of *Pythium* (Supplementary Table S2), suggesting that the increase observed in the fresh weight of *Pythium*-infested cucumber treated with strain HAI-0804 was due to the plant protection efficacy of the strain.

We also investigated the effects of strain HAI-0804 on *Pythium* damping-off and root rot at a lower inoculum intensity of pseudomonad (10^6^ CFU per ml of vermiculite). As shown in Table 3, strain HAI-0804 protected the plant but to a lesser extent than at the normal inoculum intensity (10^7^ CFU per ml of vermiculite, Table 2). The addition of glutamate (1 mM) enhanced the plant protection efficacy of the strain under this condition.

### 3.3 Strain HAI-0804 did not exhibit detectable antibiotic activity

**Figure 1:**
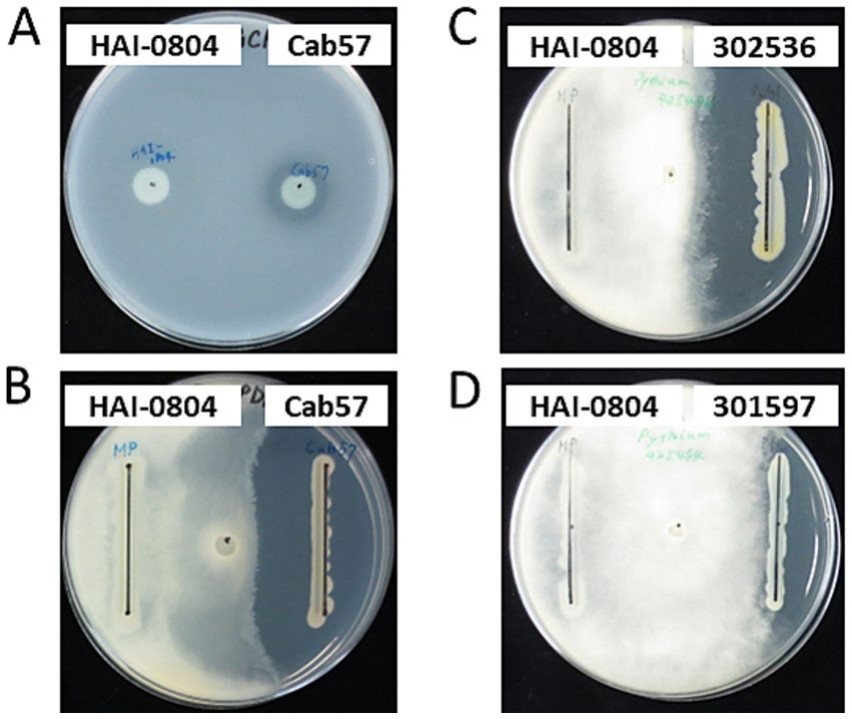
Antibiotic activities of pseudomonad strains. **(A)** The antibiotic activity of *P. rhodesiae* HAI-0804 toward *Bacillus subtilis* was compared with that of *P. protegens* Cab57 on a modified GCM plate. **(B–D)** The antibiotic activity of *P. rhodesiae* HAI-0804 toward *Pythium ultimum* was compared with that of *P. protegens* Cab57 **(B)**, with that of *P. chlororaphis* MAFF302536 **(C)**, and with that of *P. fluorescens* MAFF301597 **(D)** on PDA plates. Activities were evaluated by the size of the growth inhibition zone. The experiment was repeated three times.

To investigate whether the biocontrol activity of strain HAI-0804 was due to its antibiotic activity, we evaluated its antibiotic activities toward *B. subtilis* and *P. ultimum*. The growth of *B. subtilis* and *P. ultimum* was not inhibited by strain HAI-0804, whereas *P. protegens* Cab57 exhibited markedly strong antibiotic activity, as previously reported (Takeuchi et al., 2014). Other pseudomonad strains, such as *P. chlororaphis* and *P. fluorescens*, also exhibited strong antibiotic activity (Figure 1). These results suggest that the major contributor to the biocontrol efficacy of strain HAI-0804 was not due to an antibiotic activity under the growth medium tested. We did not try adding glutamate to the medium in this study.

### 3.4 Effects of colonization of cucumber roots by *Pseudomonas rhodesiae* HAI-0804 on the expression of defense-related genes and the production of phytohormones

To establish whether the biocontrol activity of strain HAI-0804 was due to the induction of host defenses, we examined the involvement of phytohormones by assessing the induction kinetics of SA-responsive [*pathogenesis-related* (*PR*) *protein-1*, *PR-2*, *PR-3*, *PR-4*, and *PR-5* (Pu et al., 2014; Sabbagh et al., 2018; Song et al., 2017)], JA-responsive [*lipoxygenase* (*LOX*) and *peroxidase* (*POX*); Sabbagh et al., 2018] genes, ethylene-responsive (*ETR*) (Song et al., 2017) genes, and JA/ET-responsive [phenylalanine ammonia-lyase (*PAL*); Pu et al., 2014] genes in the HAI-0804-inoculated roots of cucumber plants treated with or without glutamate (Supplementary Figure S1). The simultaneous treatment with strain HAI-0804 and glutamate exerted no or negligible effects on the expression of the nine genes. We also investigated the induction kinetics of endogenous phytohormones SA and JA. Enhancements in the accumulation of these signal compounds in the host plant were not observed with treatments by strain HAI-0804 alone, glutamate alone, or their combination (Supplementary Figure S2).

### 3.5 Glutamate affects pyoverdine production and biofilm formation by strain HAI-0804

**Figure 2:**
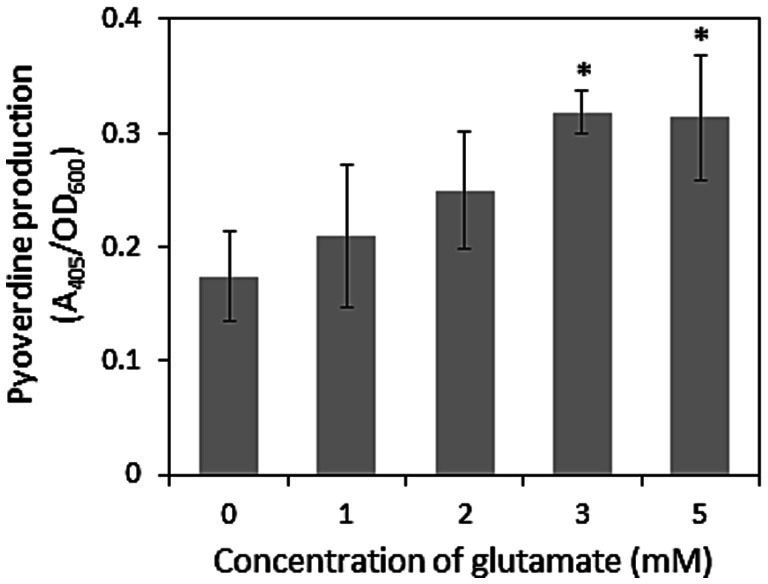
Effects of glutamate on pyoverdine production by *Pseudomonas rhodesiae* HAI-0804. Cells grown in modified GCM (liquid) with or without exogeneous glutamate (mM) were monitored. The means and standard deviations of triplicate experiments are shown. The study was repeated twice. The Dunnett’s test is used to compare each treatment with a water control and asterisks indicate values significantly different at *p* < 0.05.

Other than antibiotic activity, the siderophore production and biofilm formation abilities of pseudomonads on root surfaces are important factors for competition with other microbes in the rhizosphere (Zboralski and Filion, 2020). Rhizosphere pseudomonads generally produce the siderophore pyoverdine, which displays a high affinity for Fe(III). Therefore, we examined pyoverdine production and the effects of exogenous glutamate. As shown in Figure 2, exogenous glutamate promoted siderophore production by strain HAI-0804. These results suggest that the enhanced biocontrol activity of strain HAI-0804 by exogenous glutamate was due, at least in part, to the enhanced production of pyoverdine.

**Figure 3:**
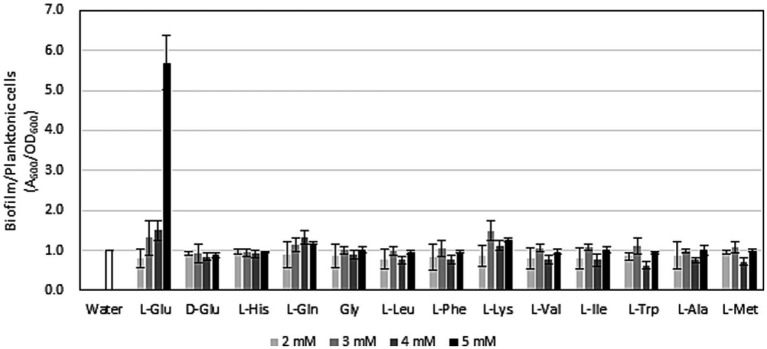
Effects of amino acids on the ratio of biofilm formation to planktonic cell growth by *Pseudomonas rhodesiae* HAI-0804. Cells grown in modified GCM (liquid) with or without exogeneous amino acids were monitored. The means and standard deviations of quadruplicate experiments of one study are shown. The study was repeated twice. Relative values are given as the water control (without amino acids) set to 1. Glu, glutamate; His, histidine; Gln, glutamine; Gly, glycine; Leu, leucine; Phe, phenylalanine; Lys, lysine; Val, valine; Ile, isoleucine; Trp, tryptophane; Ala, alanine; Met, methionine.

It currently remains unclear whether exogeneous glutamate affects biofilm formation by root-colonizing pseudomonads. The *gacA* mutant of *P. protegens* CHA0, which is a defective mutant of plant protection efficacy, exhibited a reduced ability to form biofilms *in vitro* (Takeuchi, 2018). Although this deficiency of the *gacA* mutant was mainly attributed to its inability to produce secondary metabolites, biofilm formation is important for the plant protection efficacy of root-colonizing pseudomonads (Zboralski and Filion, 2020). To confirm the effects of glutamate on biofilm formation by strain HAI-0804, glutamate was amended to the medium and the ratio of biofilm formation to planktonic cells was evaluated. As shown in Figure 3, exogenous glutamate promoted biofilm formation by strain HAI-0804 over the planktonic cell mode. To monitor the effect of the addition of glutamate on growth, strain HAI-0804 was grown under shake culture as planktonic cells. The addition of glutamate did not affect growth rates (Supplementary Figure S3). We also tested 11 other amino acids, which were examined in our previous plant protection study on *P. protegens* (Takeuchi et al., 2023b), in the L configuration and no other amino acids enhanced the ratio of biofilm formation to planktonic cells (Figure 3). Aspartate was also assessed, but it attenuated the growth of HAI-0804 under the conditions tested; therefore, we did not include these data. The D configuration of glutamate (D-Glu) did not affect biofilm formation, suggesting that the strain HAI-0804 distinguishes the DL configurations of glutamate in biofilm formation.

### 3.6 Exogenous glutamate enhanced root colonization in cucumber plants

To evaluate the function of glutamate in interactions with plants in the rhizosphere, we performed a cucumber root colonization assay with non-sterile soil to monitor a microcosm where the strain competed with other rhizosphere organisms. Strain HAI-0804 densely colonized the cucumber root even after a long-term (one month) cultivation. Exogenous glutamate increased the capacity of strain HAI-0804 for root colonization (Table 4). This result suggests that exogenous glutamate exerted positive effects on root colonization, which appeared to enhance biocontrol activity.

**Table 4:** Effect of glutamate on the ability of *Pseudomonas rhodesiae* HAI-0804 to colonize cucumber roots.

Bacterial strain and glutamate added^a^	Root fresh weight per pot (g)^b^	Colonization by *Pseudomonas rhodesiae* (log_10_ CFU per g of root)^c^
HAI-0804	0.61 ± 0.17	5.71 ± 0.12
HAI-0804 + Glu	0.73 ± 0.15	6.71 ± 0.10*

a *Pseudomonas rhodesiae* HAI-0804 was added at 10^7^ CFU per g of soil (150 mL of soil per pot) without adding *Pythium* contained within 200-ml pots, after planting three cucumber seedlings per pot. Plants were harvested after 30 days.

b Data represent the averages of three replicates per treatment.

c The rhizosphere-stable plasmid pME6031 containing a tetracycline resistance determinant was introduced as a selective marker into the bacterial strains to assess their root colonization capacities in soil.

* Denotes a value that is significantly different from that in HAI-0804 at *p* < 0.01 (*t*-test).

### 3.7 Visualization of root colonization by the strain HAI-0804 on cucumber root

**Figure 4:**
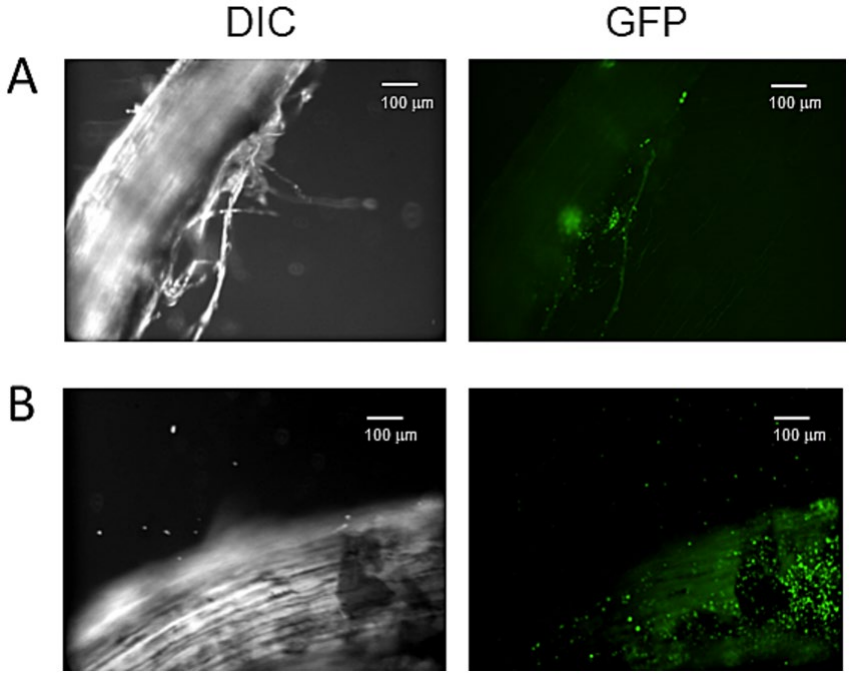
Confocal laser scanning microscopy images of a cucumber root colonized by *Pseudomonas rhodesiae* HAI-0804. Differential interference contrast (DIC) and confocal GFP images of a cucumber root colonized by *P. rhodesiae* HAI-0804 **(A,B)**. The effects of glutamate were observed in **B**. Bar = 100 μm. The experiment was repeated three times.

To visualize the effects of glutamate on the colonization of the plant root by strain HAI-0804, the cucumber root inoculated with the strain was observed under a confocal microscope. The GFP-expressing plasmid pME7402 was introduced as a marker into strain HAI-0804. As shown in Figure 4, strain HAI-0804 colonized more densely when glutamate was added, supporting the result obtained by colony counting. It is conceivable that exogenous glutamate promoted the development of biofilms on root surfaces, which conferred protection against the pathogen by building a physical barrier.

## 4 Discussion

In the present study, we demonstrated the efficacy of *P. rhodesiae* HAI-0804 to control soil-borne diseases caused by *P. ultimum*. We also showed the positive effects of glutamate on pyoverdine production, biofilm formation, root colonization, and plant protection efficacy by strain HAI-0804. Root colonization by fluorescent pseudomonads is critical for the expression of their beneficial effects (Gamalero et al., 2004). Biofilm formation following the root-colonizing process offers protection to bacteria against various stresses, such as desiccation, antibiotics, and protozoan predation, and may also promote nutrient assimilation in soil (Flemming and Wingender, 2010). Furthermore, the biofilm formation contributes to increases in the bacterial densities required to produce the secondary metabolites involved in plant–microbe and microbe–microbe interactions (Danhorn and Fuqua, 2007). Although strain HAI-0804 does not exhibit detectable antibiotic activity, the bacterial community that resides in biofilms has the advantage of niche adaptation to confer plant protection efficacy. The production of siderophores that contribute to iron deprivation in the rhizosphere may be advantageous in competition with other microorganisms. Scher and Baker (1982) showed that fluorescent pseudomonads competing for iron are responsible for the wilt-suppressiveness of the soils. They also advocated the importance of location in biocontrol. The lack of relevancy to antibiotic activity is sometimes beneficial due to their broad spectrum against pathogens because most of the antibiotic metabolites produced by pseudomonads are effective against pathogenic fungi and oomycetes, but less so against bacteria. These properties of strain HAI-0804 may contribute to its original application as a biocontrol agent against bacterial diseases on the surface of above-ground plant parts.

We previously performed an RNA-seq analysis of *P. protegens* CHA0 and the findings obtained revealed the positive effects of exogenous glutamate on chitinase gene expression of strain CHA0 (Takeuchi et al., 2023b). Although gene profiles with relevance to biofilm traits and pyoverdine production were not obtained in that study, glutamate may function as a signal that enhances attachments to root surfaces. The crucial role of biofilm formation by PGPR has been reported from many aspects in the interactions between plants and other microorganisms (Danhorn and Fuqua, 2007). In *P. protegens* CHA0, biofilm formation and colonization on plant roots by mutants with the increased production of extracellular polysaccharides were enhanced (Bianciotto et al., 2001). The importance of biofilm formation has also been reported for the Gram-positive bacterium *Bacillus subtilis*, with the surfactin-deficient mutant failing to confer protection against the pathogen (Bais et al., 2004). In *B. subtillis*, glutamate or glutamine has been reported to be essential to support biofilm formation in the synthetic medium (Hassanov et al., 2018).

Regarding the kinetics of the induction of systemic resistance in plants, root-colonizing pseudomonads were previously reported to induce systemic resistance in ET- and JA-dependent manners in studies on *Arabidopsis* utilizing mutants with impaired plant hormone signaling (Iavicoli et al., 2003; Ahn et al., 2007). The concentrations of endogenous glutamate in plants were reported to be at a low micromolar level and reached approximately 50 mM at damaged leaves (Toyota et al., 2018). Assuming similar conditions in natural roots, glutamate released from roots damaged by pathogens may trigger the beneficial function of root-colonizing pseudomonads. Exogenous glutamate has also been shown to induce the expression of defense-related genes, as revealed by transcriptome analyses of *Arabidopsis* (Goto et al., 2020). In the present study, the results shown in Supplementary Figures S1, S2 suggest that the induction of systemic resistance is not a major factor contributing to resistance against *Pythium* damping-off in cucumber under the conditions tested, although the possibility of a priming effect could not be excluded. Since the enhancement of biocontrol efficacy by exogenous glutamate correlates with the production of the siderophore pyoverdine, root colonization in cucumber plants, and the ratio of biofilm formation, these factors make a significant contribution to biocontrol efficacy against *Pythium* damping-off in cucumber. Considering the fact that glutamate is one of the building blocks of pyoverdine (Ringel and Brüser, 2018), it is plausible that the exogenous glutamate contributed to the increased level of pyoverdine production. Glutamate has also been reported to be one of the major amino acid components of root exudate in tomato (Simons et al., 1997). In that study, amino acid synthesis has been shown to be necessary for root colonization by fluorescent pseudomonads. In this context, there is a precedent that deserves to be mentioned: the colonization by pseudomonads changes the composition of root exudate (Zhang et al., 2023). Therefore, we also consider that cucumber root exudate affected by the colonization by HAI-0804 could alter the growth of *Pythium* in our present study. Another possible mechanism behind is a model based on the type VI secretion systems. Although the system has been found in bacterial interactions, the recently discovered Type VI secretion effectors against fungal cells (Trunk et al., 2018) remind us of the involvement in *Pythium* control by HAI-0804.

In addition to the application of the beneficial strains themselves, systematic control, which favors the functions of the strains by designing suitable chemical properties for the rhizosphere, may be an effective approach to improve biocontrol efficacy. D-amino acids isolated from the cell walls of bacteria have been reported to trigger the disassembly of biofilms in *B. subtilis*, *P. aeruginosa*, and *Staphylococcus aureus* (Leiman et al., 2013; Sanchez et al., 2014), whereas D-Glu did not affect the formation of biofilms on strain HAI-0804 in the present study. Not only biofilm building-up processes but also biofilm dispersal mechanisms are of interest because the dispersal process allows bacteria to expand their residence in the rhizosphere. This aspect will be more critical for the long-term cultivation of plants under conditions where the bacterial life cycle of biofilms and planktonic modes is repeated. In this study, we have revealed the positive effect of glutamate on the performance of HAI-0804. By utilizing these inexpensive amino acids properly, it will be possible to control the switch of PGPR from planktonic to sessile growth and vice versa for the fine regulation of rhizosphere colonization.

## Data Availability

The 16S rRNA gene sequence of strain HAI-0804 has been deposited in the DDBJ/ENA/GenBank database under accession no. DL243255.
