# Supplementary material for: Pseudomonas rhodesiae HAI-0804 suppresses Pythium damping off and root rot in cucumber by its efficient root colonization promoted by amendment with glutamate
Source: Front Microbiol. 2024 Nov 5;15:1485167. doi: 10.3389/fmicb.2024.1485167 (PMC11573540; doi:10.3389/fmicb.2024.1485167)
Supplement: Supplementary file 1 [file Data_Sheet_1.docx]

Supplementary Material

## Supplementary Figures

##

## Supplementary Figure 1. Gene expression analysis of cucumber plants. The roots of cucumber plants were inoculated with HAI-0804 (termed MP) with or without glutamate and then incubated. Roots were harvested at the indicated time intervals after inoculation and used for total RNA extraction followed by a quantitative real-time PCR analysis. The roots of cucumber plants that were treated with H_2_O alone, but not inoculated with HAI-0804 (MP), were used as a control. Values are the mean ± SD (n = three biological replicates). Asterisks denote significant differences from the H_2_O sample (*P* < 0.05).

**Supplementary Figure 2.** Phytohormone analysis of cucumber plants. The roots of cucumber plants were inoculated with HAI-0804 (termed MP) with or without glutamate and then incubated. Roots were harvested at the indicated time intervals after the inoculation and used for measurements of defense-related substances. The roots of cucumber plants that were treated with H_2_O alone, but not inoculated with HAI-0804 (MP), were used as a control. Values are the mean ± SD (n = three biological replicates).

**Supplementary Figure 3.** The growth of *Pseudomonas rhodesiae* HAI-0804 in modified GCM (liquid) with or without exogeneous glutamate was monitored. Strain HAI-0804 was grown in Erlenmeyer flasks at 180 rpm at 28°C after the inoculation (scaling up from an overnight culture to a fresh culture, 100 times). Measurements were performed in triplicate. Symbols indicate averages, and error bars indicate S.D. values. OD_600nm_, optical density at 600 nm.

## Supplementary Tables

**Supplementary Table 1**. List of primers used in quantitative real-time PCR.

| Gene name | Accession number | Primer sequence (5'–3') Forward/Reverse |
| --- | --- | --- |
| *CsPR1* | XM_011660558 | TGCTCAACAATATGCGAACC  TCATCCACCCACAACTGAAC |
| *CsPR2* | XM_011657726 | GGGCGGAGTCAAGAGAGCGT  TGCCACCACATCTGACCTGCT |
| *CsPR3* | XM_004151681 | TGGTCACTGCAACCCTGACA  AGTGGCCTGGAATCCGACT |
| *CsPR4* | XM_004139193 | GCCCTTGAATTGGCGCAAACAC  TTCGACCTGATGGGCCGACG |
| *CsPR5* | XM_004137802 | TTAACGAGCGGTCCAGGCCA  ACCGCAATCGCCAGTCATGC |
| *CsLOX* | XM_031880629 | GAGAGCGTAAGGAATGGGATAGAA  CACCGGGTTCGGAAAGG |
| *CsETR* | KP119741 | GCCATTGTTGCAAAAGCAGA  GCCAAAGACCACTGCCACA |
| *CsPAL* | XM_031887954 | ATGGAGGCAACTTCCAAGGA  CCATGGCAATCTCAGCACCT |
| *CsPOX* | XM_011651657 | AGACGCCGAGAAGGACGCTC  TCGGCGCAAGAGACTCGACC |
| *CsCACS* | GW881874 | TGGGAAGATTCTTATGAAGTGC  CTCGTCAAATTTACACATTGGT |

**Supplementary Table 2.** Evaluation of the fresh weight of cucumber with each treatment.

| Bacterial strain  and glutamate added* | *Pythium* added | Surviving plants per pot (%)** | Shoot fresh weight per pot (g)** | Root fresh weight per pot (g)** |
| --- | --- | --- | --- | --- |
| None | - | 100 ^a^ | 1.16 ^a^ | 0.38 ^a^ |
| HAI-0804 | - | 100 ^a^ | 1.26 ^a^ | 0.41 ^a^ |
| HAI-0804 + Glu1 | - | 100 ^a^ | 1.20 ^a^ | 0.37 ^a^ |
| HAI-0804 + Glu10 | - | 100 ^a^ | 1.29 ^a^ | 0.36 ^a^ |

* *P. rhodesiae* HAI-0804 was added at 10^7^ CFU per gram of vermiculite (25 g of vermiculite per pot) contained within 125-ml pots, after planting three cucumber seedlings per pot. Plants were harvested after 14 days.

** Data represent the averages of 12 replicates (from two independent experiments) per treatment.
